# Tandem Aldol-Michael Reactions in Aqueous Diethylamine Medium: A Greener and Efficient Approach to Bis-Pyrimidine Derivatives

**DOI:** 10.3390/ijms141223762

**Published:** 2013-12-05

**Authors:** Abdullah M. Al-Majid, Assem Barakat, Hany J. AL-Najjar, Yahia N. Mabkhot, Hazem A. Ghabbour, Hoong-Kun Fun

**Affiliations:** 1 Department of Chemistry, Faculty of Science, King Saud University, P.O. Box 2455, Riyadh 11451, Saudi Arabia; E-Mails: amajid@ksu.edu.sa (A.M.A.-M.); hany_33@hotmail.com (H.J.A.-N.); yahia@ksu.edu.sa (Y.N.M.); 2 Department of Chemistry, Faculty of Science, Alexandria University, P.O. Box 426-Ibrahimia, Alexandria 21321, Egypt; 3 Department of Pharmaceutical Chemistry, Faculty of Pharmacy, King Saud University, P.O. Box 2457, Riyadh 11451, Saudi Arabia; E-Mails: ghabbourh@yahoo.com (H.A.G.); hfun.c@ksu.edu.sa (H.-K.F.)

**Keywords:** tandem Aldol-Michael reactions, MCRs, barbituric acid, aqueous media, green chemistry

## Abstract

A simple protocol, involving the green synthesis for the construction of novel bis-pyrimidine derivatives, **3a**–**i** and **4a**–**e** are accomplished by the aqueous diethylamine media promoted tandem Aldol-Michael reaction between two molecules of barbituric acid derivatives **1a**,**b** with various aldehydes. This efficient synthetic protocol using an economic and environmentally friendly reaction media with versatility and shorter reaction time provides bis-pyrimidine derivatives with high yields (88%–99%).

## 1. Introduction

**Figure 1.**
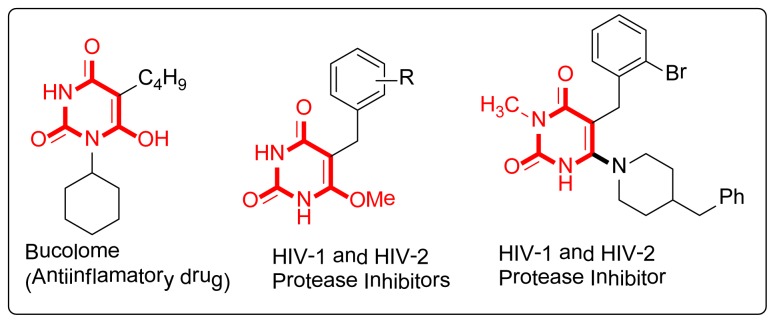
Bioactive compounds containing the barbituric acid framework.

Derivatives of the 1,3-dimethylbarbituric acid (**1**) framework represent an important structural motif that is embodied in a number of bioactive natural products [1–5]. In particular, 5-Alkylated barbituric acids are one class of such compounds that possess diverse biological activity, with anticancer, HIV-1 and HIV-2 protease inhibitors [6], sedative-hypnotic [7,8], and anticonvulsant [9] properties. Many of their representatives have clinical use as antiinflammatory and hypnotic drugs, such as bucolome (Figure 1), veronal, phenobarbital, seconal, and sodium pentothal [10–13]. A number of compounds having these systems are synthesized with diverse pharmacological activity [14,15]. New routes for the synthesis of these molecules have therefore attracted considerable attention in the search for a rapid entry to these heterocycles with diverse biological properties. In accordance with the principles of green chemistry, the design of easily separable, non-toxic and low cost synthesis obviating the isolation and purification of intermediates and thus leading to a reduction in pollution has become an important area of research in chemistry [16]. A potential approach toward this goal is to combine two or more distinct reactions into a single transformation, thereby affecting tandem reactions [17–27]. Tandem reactions are therefore of increasing importance in modern organic chemistry. One-pot tandem reactions by virtue of their convergence, elegance, and super-economy are particularly appealing in the context of rapid target-oriented synthesis [28–30].

We have investigated a new, simple and efficient synthesis of novel bis-pyrimidines derivatives based on tandem Aldol-Michael reactions using aqueous diethylamine medium.

## 2. Results and Discussion

**Figure 2.**
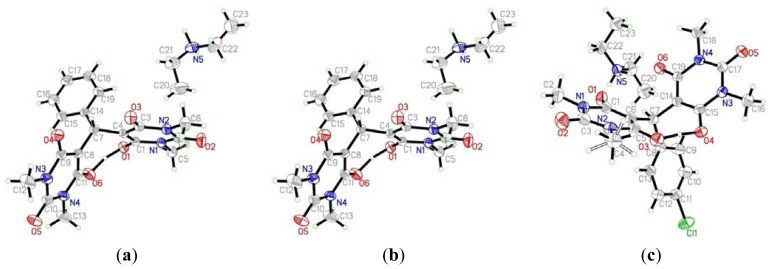
ORTEP representation of the structure of **3a**–**c**.

Upon treatment of 2 molecules from 1,3-dimethylbarbituric acid **1a**, with benzaldehyde **2a**, water (1.5 mL) and in presence of one equivalent diethylamine, a one-pot three-component reaction proceeded spontaneously at room temperature. “Trimolecular adduct salts” **3a** was obtained in 99% yield by simple filtration. The structure of the compound **3a** was elucidated using spectroscopic data and elemental analyses. The NMR spectra confirmed the absence of the methylene proton of the 1,3-dimethylbarbituric acid moiety and the presence of a proton at *δ* 5.86 detected ppm revealing it to be the benzylic proton. The 3D structure was confirmed by single crystal structure determination (Figure 2a).

Encouraged by this result, we proceeded to study the effect of different amines and reaction conditions on the tandem Aldol-Michael reactions for the synthesis of bis-pyrimidine derivatives.

Due to the fact that the aqueous diethylamine medium gave the desired product **3a** with quantitative yield, several secondary amines were therefore tested. Compared to aqueous diethylamine, aqueous diisopropylamine, aqueous dicyclohexylamine, and aqueous morpholine, which contain a bulkier amine, also gave the product but with less yield and lower reaction rates (Table 1, entries 2–4 respectively). Furthermore, NaOH was also examined and was found to be less efficient in the reaction. Only moderate yield was obtained (Table 1, entry 5). We also found that in the absence of water (entry 6) or with only water as a reactant (entry 7), the reaction either could not be processed or preceded very slowly.

The X-ray structure of diethylaminium salt **3b** (Figure 2b) was obtained from a single crystal grown from DCM/Et_2_O as a solvent, and the structure shows interesting characteristics. We were unable to determine the location of the C4 and C8 hydrogens by ^1^H-NMR analysis. The hydrogen from C4, rather than from C8 of the barbituric acid moiety, is removed by the basicity of diethylamine. We determined that this was due to the fact that one hydrogen is on the diethylamine and the other is involved in hydrogen bonding interactions between both barbituric acid rings. It is noteworthy to mention that ^1^H-NMR also have shown a singlet signal at *δ* 17.64 pmm, which was revealed to belong to the OH group that is making a hydrogen bond. This hypothesis was further unambiguously confirmed by the elucidation the 3D chemical structure of product **3c** by single crystal X-ray crystallography (Figure 2c).

**Figure 3.**
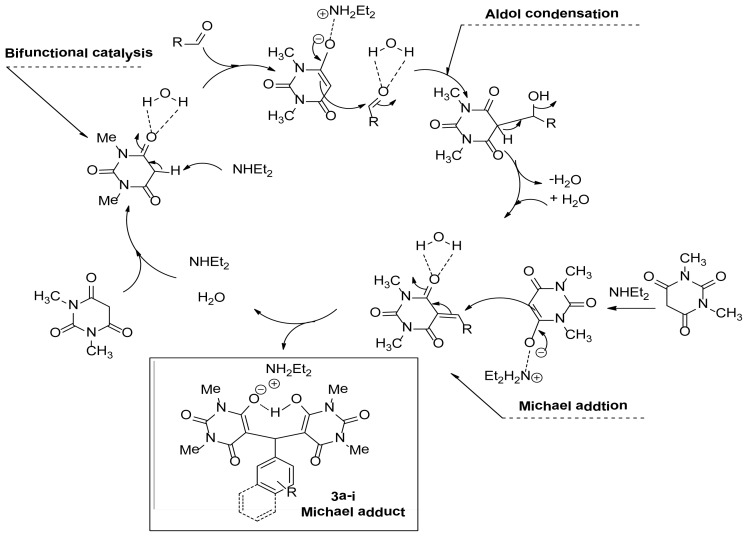
A possible mechanistic pathway.

A possible mechanism for the tandem Aldol-Michael reaction is shown in Figure 3. First, the hydrogen bonding activation of the C=O group by water eases the deprotonation of **1a** by diethylamine. The aldehyde is then attacked by the enolate to form the Aldol condensation product after dehydration of the intermediate. The second Michael addition under these conditions produces the final product **3** (Figure 3) [31–37].

With the optimal reaction conditions established, the generality of the Aldol-Michael reactions was next investigated by using a series of aldehydes (Table 2). Various aldehydes derivatives with either electron-withdrawing or electron-donating groups at the para-, meta-, or even sterically hindered ortho-position on the aromatic ring were tolerated and gave the corresponding bis-pyrimidine derivatives **3a**–**i** in excellent chemical yield up to 99% (Table 2). In addition, reactions with substrates **2**, which contain sterically hindered 1-naphthyl proceeded smoothly to give products with very good results (Table 2, entry 9).

Finally, to confirm the general applicability of this novel reaction method in the preparation of a wide variety of bis-pyrimidines derivatives, we used barbituric acid itself. In every case, the corresponding bis-pyrimidines derivatives **4a**–**e** were isolated in excellent quantitative yields (Table 3).

## 3. Experimental Section

General: Chemicals were purchased from Sigma-Aldrich, Fluka (Seelze, Germany), and were used without further purification, unless otherwise stated. Melting points were measured on a Gallenkamp melting point apparatus (Weiss Gallenkamp Ltd., Leicestershire, UK) in open glass capillaries and are uncorrected. IR Spectra were measured as KBr pellets on a Nicolet 6700 FT-IR spectrophotometer (Madison, WI, USA). The NMR spectra were recorded on a Varian Mercury Jeol-400 NMR spectrometer (Tokyo, Japan).^1^H-NMR (400 MHz), and ^13^C-NMR (100 MHz) were run in either deuterated dimethylsulphoxide (DMSO-*d*_6_) or deuterated chloroform (CDCl_3_). Chemical shifts (*δ*) are referred in terms of ppm and *J*-coupling constants are given in Hz. Mass spectra were recorded on a Jeol of JMS-600 H (Tokyo, Japan). Elemental analysis was carried out on an Elmer 2400 elemental analyzer, CHN mode (Vernon Hills, IL, USA).

### 3.1. General Procedure for Aldol Condensation Michael Addition for the Synthesis of **3a**–**i** and **4a**–**e** (GP1)

A mixture of aldehyde **2** (1.5mmol), barbituric acid derivatives **1a**,**b** (3 mmol) and Et_2_NH (1.5 mmol, 155 μL) in 3 mL of degassed H_2_O (bubbling nitrogen through the water) was stirred at room temperature for 1–5 h until TLC showed complete disappearance of the reactants. The precipitate was removed by filtration and washed with ether (3 × 20 mL). The solid was dried to afford pure product **3a**–**i** and **4a**–**e**.

#### 3.1.1. 5,5′-(Phenylmethylene)bis(1,3-dimethylpyrimidine-2,4,6(1*H*,3*H*,5*H*)-trione) Diethylaminium Salt **3a**

**3a** was prepared from 1,3-dimethylbarbituric acid **1a**, and benzaldehyde **2a** according to the general procedure (**GP1**) yielding colorless crystalline materials (1.4 g, 2.97 mmol, 99%). m.p.: 192 °C; IR (KBr, cm^−1^): 3450, 3222, 3019, 2814, 1707, 1655, 1524, 1442, 1345, 1274; ^1^H-NMR (400 MHz, DMSO-*d*_6_): *δ* 17.64 (s, 1H, OH), 7.58 (d, 2H, *J* = 7.3 Hz, NH_2_), 7.19 (m, 5H, Ph), 5.86 (s, 1H, benzyl-H), 3.05 (s, 12H, 4CH_3_), 2.93 (q, 4H, *J* = 7.3 Hz, C*H*_2_CH_3_), 1.16 (t, 6H, *J* = 7.3 Hz, CH_2_C*H*_3_); ^13^C-NMR (100 MHz, DMSO-*d*_6_): *δ* = 161.6, 153.2, 145.5, 141.6, 129.1, 128.2, 127.8, 125.8, 88.5, 49.1, 41.9, 27.5, 11.5; LC/MS (ESI): 473 [M]^+^; Anal. for C_23_H_31_N_5_O_6_; Calcd: C, 58.34; H, 6.60; N, 14.79; Found: C, 58.35; H, 6.62; N, 14.80.

The structure of **3a** was confirmed by X-ray crystal structure analysis (Bruker AXS GmbH, Karlsruhe, Germany). CCDC-933457 contains the supplementary crystallographic data for this compound. These data can be obtained free of charge from the Cambridge Crystallographic Data Centre via http://www.ccdc.cam.ac.uk/data_request/cif. A colorless crystal suitable for X-ray analysis was obtained from recrystallization of the compound from DCM/Et_2_O at room temperature after 5 days.

#### 3.1.2. 5,5′-(*p*-Tolylmethylene)bis(1,3-dimethylpyrimidine-2,4,6(1*H*,3*H*,5*H*)-trione) Diethylaminium Salt **3b**

**3b** was prepared from 1,3-dimethylbarbituric acid **1a**, and *p*-tolualdehyde **2b** according to the general procedure (**GP1**) yielding colorless needle materials (1.41 g, 2.91 mmol, 97%). m.p.: 152 °C; IR (KBr, cm^−1^): 3455, 3210, 2984, 2820, 1560, 1449, 1359; ^1^H-NMR (400 MHz, CDCl_3_): *δ* 17.64 (s, 1H, OH), 6.99–6.96 (m, 4H, Ph), 5.80 (s, 1H, benzyl-H), 3.32 (s, 12H, 4CH_3_), 3.03 (q, 4H, *J* = 7.3 Hz, C*H*_2_CH_3_), 2.25 (s, 3H, CH_3_), 1.28 (t, 6H, *J* = 7.3 Hz, CH_2_C*H*_3_); ^13^C-NMR (100 MHz, CDCl_3_): *δ* = 165.3, 164.3, 151.8, 138.6, 134.8, 128.9, 126.3, 92.1, 42.0, 34.2, 28.9, 28.6, 21.0, 11.4; LC/MS (ESI): 487[M]^+^; Anal. for C_24_H_35_N_5_O_6_; Calcd: C, 59.12; H, 6.82; N, 14.36; Found: C, 59.13; H, 6.81; N, 14.35.

The structure of **3b** was confirmed by X-ray crystal structure analysis (Bruker AXS GmbH). CCDC-957025 contains the supplementary crystallographic data for this compound. These data can be obtained free of charge from the Cambridge Crystallographic Data Centre via www.ccdc.cam.ac.uk/data_request/cif. A colorless crystal suitable for X-ray analysis was obtained from recrystallization the compound from DCM/Et_2_O at room temperature after 2 days.

#### 3.1.3. 5,5′-((4-Chlorophenyl)methylene)bis(1,3-dimethylpyrimidine-2,4,6(1*H*,3*H*,5*H*)-trione) Diethylaminium Salt **3c**

**3c** was prepared from 1,3-dimethylbarbituric acid **1a**, and *p*-chlorobenzaldehyde **2c** according to the general procedure (**GP1**) yielding colorless crystals (1.44 g, 2.85 mmol, 95%). m.p.: 103 °C; IR (KBr, cm^−1^): 3450, 3198, 2988, 1698, 1603, 1479, 1385; ^1^H-NMR (400 MHz, CDCl_3_): *δ* 17.66 (s, 1H, OH), 7.18 (d, 2H, *J* = 8.8 Hz, Ph), 7.05 (d, 2H, *J* = 8.8 Hz, Ph), 5.80 (s, 1H, benzyl-H), 3.34 (s, 12H, 4CH_3_), 3.06 (q, 4H, *J* = 7.3 Hz, C*H*_2_CH_3_), 1.30 (t, 6H, *J* = 7.3 Hz, CH_2_C*H*_3_); ^13^C-NMR (100 MHz, CDCl_3_): *δ* = 165.3, 164.3, 151.7, 138.6, 134.8, 128.2, 126.3, 91.7, 42.1, 34.2, 28.9, 28.7, 11.5; LC/MS (ESI): 507[M]^+^; Anal. for C_23_H_30_ClN_5_O_6_; Calcd: C, 54.38; H, 5.95; Cl, 6.98; N, 13.79; Found: C, 54.38; H, 5.94; Cl, 7.01; N, 13.81.

The structure of **3c** was confirmed by X-ray crystal structure analysis (Bruker AXS GmbH). CCDC-957026 contains the supplementary crystallographic data for this compound. These data can be obtained free of charge from the Cambridge Crystallographic Data Centre via www.ccdc.cam.ac.uk/data_request/cif. A colorless crystal suitable for X-ray analysis was obtained from recrystallization the compound from DCM/Et_2_O at room temperature after 2 days.

#### 3.1.4. 5,5′-((4-Bromophenyl)methylene)bis(1,3-dimethylpyrimidine-2,4,6(1*H*,3*H*,5*H*)-trione) Diethylaminium Salt **3d**

**3d** was prepared from 1,3-dimethylbarbituric acid **1a**, and *p*-bromobenzaldehyde **2d** according to the general procedure (**GP1**) yielding beige powder (1.5 g, 2.76 mmol, 92%). m.p.: 125 °C; IR (KBr, cm^−1^): 3454, 3200, 3019, 2814, 1707, 1650, 1518, 1442, 1377, 1274; ^1^H-NMR (400 MHz, CDCl_3_): *δ* 17.62 (s, 1H, OH), 7.31 (d, 2H, *J* = 8.8 Hz, Ph), 6.99 (d, 2H, *J* = 8.8 Hz, Ph), 5.79 (s, 1H, benzyl-H), 3.33 (s, 12H, 4CH_3_), 3.03 (q, 4H, *J* = 7.3 Hz, C*H*_2_CH_3_), 1.27 (t, 6H, *J* = 7.3 Hz, CH_2_C*H*_3_) ; ^13^C-NMR (100 MHz, CDCl_3_): *δ* = 165.3, 164.3, 151.7, 141.1, 131.1, 128.4, 119.3, 91.7, 42.1, 34.2, 28.9, 28.7, 11.4; LC/MS (ESI): 552[M]^+^; Anal. for C_23_H_30_BrN_5_O_6_; Calcd: C, 50.01; H, 5.47; Br, 14.46; N, 12.68; Found: C, 50.00; H, 5.47; Br, 14.49; N, 12.70.

#### 3.1.5. 5,5′-((3-Bromophenyl)methylene)bis(1,3-dimethylpyrimidine-2,4,6(1*H*,3*H*,5*H*)-trione) Diethylaminium Salt **3e**

**3e** was prepared from 1,3-dimethylbarbutric acid **1a**, and *m*-bromobenzaldehyde **2e** according to the general procedure (**GP1**) yielding colorless crystalline materials (1.5g, 2.76 mmol, 92%). m.p.: 169 °C; IR (KBr, cm^−1^): 3450, 3120, 2982, 1694, 1667, 1615, 1577, 1445, 1250; ^1^H-NMR (400 MHz, CDCl_3_): *δ* 17.63 (s, 1H, OH), 7.22 (d, 1H, *J* = 7.3 Hz, Ph), 7.19 (s, 1H, Ph), 7.07 (d, 1H, *J* = 7.3 Hz, Ph), 7.05 (d, 1H, *J* = 7.3 Hz, Ph), 5.84 (s, 1H, benzyl-H), 3.34 (s, 6H, 2CH_3_), 3.32 (s, 6H, 2CH_3_), 3.02 (q, 4H, *J* = 7.3 Hz, C*H*_2_CH_3_), 1.27 (t, 6H, *J* = 7.3 Hz, CH_2_C*H*_3_); ^13^C-NMR (100 MHz, CDCl_3_): *δ* = 165.2, 164.4, 151.7, 144.7, 129.7,129.6, 128.7, 125.3, 91.5, 42.1, 34.4, 28.9, 28.7, 11.5; LC/MS (ESI): 552[M]^+^; Anal. for C_23_H_30_BrN_5_O_6_; Calcd: C, 50.01; H, 5.47; Br, 14.46; N, 12.68; Found: C, 50.03; H, 5.48; Br, 14.47; N, 12.71.

#### 3.1.6. 5,5′-((4-Methoxyphenyl)methylene)bis(1,3-dimethylpyrimidine-2,4,6(1*H*,3*H*,5*H*)-trione) Diethylaminium Salt **3f**

**3f** was prepared from 1,3-dimethylbarbutric acid **1a**, and *p*-methoxybenzaldehyde **2f** according to the general procedure (**GP1**) yielding rose-colored crystalline materials (1.35 g, 2.7 mmol, 90%). m.p.: 160 °C; IR (KBr, cm^−1^): 3445, 3195, 2977, 2836, 1689, 1664, 1613, 1504, 1447, 1378, 1242; ^1^H-NMR (400 MHz, CDCl_3_): *δ* 17.67 (s, 1H, OH), 7.01 (d, 2H, *J* = 8.8 Hz, Ph), 6.75 (d, 2H, *J* = 8.8 Hz, Ph), 5.79 (s, 1H, benzyl-H), 3.33 (s, 12H, 4CH_3_), 2.99 (q, 4H, *J* = 7.3 Hz, C*H*_2_CH_3_), 1.26 (t, 6H, *J* = 7.3 Hz, CH_2_C*H*_3_); ^13^C-NMR (100 MHz, CDCl_3_): *δ* = 165.3, 164.3, 157.4, 151.7, 133.6, 132.0, 127.4, 114.3, 92.1, 55.6, 42.1, 33.8, 28.9, 11.5; LC/MS (ESI): 503[M]^+^; Anal. for C_24_H_33_N_5_O_7_; Calcd: C, 57.25; H, 6.61; N, 13.91; Found: C, 57.26; H, 6.61; N, 13.90.

#### 3.1.7. 5,5′-((4-Nitrophenyl)methylene)bis(1,3-dimethylpyrimidine-2,4,6(1*H*,3*H*,5*H*)-trione) Diethylaminium Salt **3g**

**3g** was prepared from 1,3-dimethylbarbutric acid **1a**, and *p*-nitrobenzaldehyde **2g** according to the general procedure (**GP1**) yielding a yellow powder (1.35 g, 2.61 mmol, 87%); m.p.: 195 °C; IR (KBr, cm^−1^): 3453, 3205, 2987, 2904, 1675, 1608, 1576, 1511, 1438, 1343, 1254; ^1^H-NMR (400 MHz, CDCl_3_): *δ* 17.58 (s, 1H, OH), 8.08 (d, 2H, *J* = 8.8 Hz, Ph), 7.29 (d, 2H, *J* = 8.8 Hz, Ph), 5.95 (s, 1H, benzyl-H), 3.34 (s, 12H, 4CH_3_), 3.07 (q, 4H, *J* = 7.3 Hz, C*H*_2_CH_3_), 1.29 (t, 6H, *J* = 7.3 Hz, CH_2_C*H*_3_); ^13^C-NMR (100 MHz, CDCl_3_): *δ* = 165.2, 164.4, 151.6, 150.8, 146.1, 127.5, 123.5, 91.4, 42.2, 34.9, 28.9, 28.7, 11.5; LC/MS (ESI): 518[M]^+^; Anal. for C_23_H_30_N_6_O_8_; Calcd: C, 53.28; H, 5.83; N, 16.21; Found: C, 53.29; H, 5.85; N, 16.23.

#### 3.1.8. 5,5′-(3-Tolylmethylene)bis(1,3-dimethylpyrimidine-2,4,6(1*H*,3*H*,5*H*)-trione) Diethylaminium Salt **3h**

**3h** was prepared from 1,3-dimethylbarbituric acid **1a**, and *m*-tolualdehyde **2h** according to the general procedure (**GP1**) yielding rose-colored crystalline materials. (1.41 g, 2.91 mmol, 97%). m.p.: 135 °C; IR (KBr, cm^−1^): 3455, 3201, 2988, 1693, 1667, 1611, 1573, 1443; ^1^H-NMR (400 MHz, CDCl_3_): *δ* 17.62 (s, 1H, OH), 7.10 (t, 1H, *J* = 7.3 Hz, Ph), 6.92 (d, 1H, *J* = 7.3 Hz, Ph), 6.88 (d, 1H, *J* = 7.3 Hz, Ph), 5.82 (s, 1H, benzyl-H), 3.32 (s, 12H, 4CH_3_), 3.01 (q, 4H, *J* = 7.3 Hz, C*H*_2_CH_3_), 2.25 (s, 3H, CH_3_), 1.26 (t, 6H, *J* = 7.3 Hz, CH_2_C*H*_3_); ^13^C-NMR (100 MHz, CDCl_3_): *δ* = 165.3, 164.4, 151.8, 141.7, 137.4, 127.9, 127.1, 126.4, 123.6, 92.1, 42.0, 34.4, 28.9, 28.6, 21.8, 11.4; LC/MS (ESI): 487[M]^+^; Anal. for C_24_H_35_N_5_O_6_; Calcd: C, 59.12; H, 6.82; N, 14.36; Found: C, 59.13; H, 6.81; N, 14.35.

#### 3.1.9. 5,5′-(Naphthalen-2-ylmethylene)bis(1,3-dimethylpyrimidine-2,4,6(1*H*,3*H*,5*H*)-trione) Diethylaminium Salt **3i**

**3i** was prepared from 1,3-dimethylbarbutric acid **1a**, and 2-naphthaldehyde **2i** according to the general procedure (**GP1**) yielding beige powder (1.47 g, 2.82 mmol, 94%). m.p.: 146 °C; IR (KBr, cm^−1^): 3454, 3200, 2967, 1668, 1585, 1438, 1250; ^1^H-NMR (400 MHz, CDCl_3_): *δ* 17.33 (s, 1H, OH), 8.10 (d, 2H, *J* = 8.8 Hz, naphthyl-H), 7.99 (d, 2H, *J* = 8.8 Hz, naphthyl-H), 7.92 (d, 2H, *J* = 8.8 Hz, naphthyl-H), 7.90 (d, 2H, *J* = 8.8 Hz, naphthyl-H), 7.84 (d, 2H, *J* = 8.8 Hz, naphthyl-H), 7.68–7.38 (m, 3H, naphthyl-H), 6.37 (s, 1H, benzyl-H), 3.39 (s, 12H, 4CH_3_), 3.01 (q, 4H, *J* = 7.3 Hz, C*H*_2_CH_3_), 1.30 (t, 6H, *J* = 7.3 Hz, CH_2_C*H*_3_); ^13^C-NMR (100 MHz, CDCl_3_): *δ* = 164.9, 151.7, 136.8, 135.3, 134.3, 131.5, 129.1, 128.5, 127.0, 125.2 124.9, 123.8, 93.2, 41.8, 33.2, 28.8, 11.4; LC/MS (ESI): 523 [M]^+^; Anal. for C_27_H_33_N_5_O_6_; Calcd: C, 61.94; H, 6.35; N, 13.38; Found: C, 61.95; H, 6.34; N, 13.40.

#### 3.1.10. 5,5′-(Phenylmethylene)bis(6-hydroxypyrimidine-2,4(1*H*,3*H*)-dione) Diethylaminium Salt **4a**

**4a** was prepared from barbituric acid **1b**, and benzaldehyde **2a** according to the general procedure (**GP1**) yielding an oil product (1.22g, 2.94mmol, 98%); IR (KBr, cm^−1^): 3450, 3200, 2976, 2839, 1668, 1615, 1505, 1383, 1247; ^1^H-NMR (400 MHz, DMSO-*d_6_*): δ 17.22 (s, 1H, OH), 10.10 (bs, 4H, NH), 7.14 (t, 3H, *J* = 7.3 Hz, Ph), (d, 2H, *J* = 7.3 Hz, Ph), 7.29 (d, 2H, *J* = 8.8 Hz, Ph), 5.95 (s, 1H, benzyl-H), 2.93 (q, 4H, *J* = 7.3 Hz, C*H*_2_CH_3_), 1.15 (t, 6H, *J* = 7.3 Hz, CH_2_C*H*_3_); ^13^C-NMR (100 MHz, DMSO-*d_6_*): δ = 165.3, 164.4, 151.7, 142.1.3, 134.3, 129.9, 127.3, 91.6, 42.6, 30.1, 11.6; LC/MS (ESI): 417[M]^+^; Anal. for C_19_H_23_N_5_O_6_; Calcd: C, 54.67; H, 5.55; N, 16.78; Found: C, 54.68; H, 5.54; N, 16.79.

#### 3.1.11. 5,5′-(*p*-Tolylmethylene)bis(6-hydroxypyrimidine-2,4(1*H*,3*H*)-dione) Diethylaminium Salt **4b**

**4b** was prepared from barbituric acid **1b**, and *p*-tolualdehyde **2b** according to the general procedure (**GP1**) yielding white powder (1.22 g, 2.85 mmol, 95%); m.p.: 205 C; IR (KBr, cm^−1^): 3459, 3120, 2978, 2811, 1689, 1612, 1325, 1252; ^1^H-NMR (400 MHz, DMSO-*d_6_*): *δ* 17.18 (s, 1H, OH), 10.09 (bs, 4H, NH), 6.93 (m, 4H, Ph), 5.90 (s, 1H, benzyl-H), 2.79 (q, 4H, *J* = 7.3 Hz, C*H*_2_CH_3_), 2.20 (s, 3H, CH_3_), 1.07 (t, 6H, *J* = 7.3 Hz, CH_2_C*H*_3_); ^13^C-NMR (100 MHz, DMSO-*d_6_*): *δ* = 164.8, 164.1, 151.3, 142.1, 133.5, 128.5, 127.1, 91.6, 42.6, 30.6, 21.1, 13.0; LC/MS (ESI): 431[M]^+^; Anal. for C_20_H_25_N_5_O_6_; Calcd: C, 55.68; H, 5.84; N, 16.23; Found: C, 55.67; H, 5.83; N, 16.22.

#### 3.1.12. 5,5′-((4-Chlorophenyl)methylene)bis(6-hydroxypyrimidine-2,4(1*H*,3*H*)-dione) Diethylaminium Salt **4c**

**4c** was prepared from barbituric acid **1b**, and *p*-chlorobenzaldehyde **2c** according to the general procedure (**GP1**) yielding a white powder (1.28 g, 2.85 mmol, 95%); m.p.: 221 °C; IR (KBr, cm^−1^): 3435, 3185, 2978, 2830, 1677, 1548, 1448, 1345, 1250; ^1^H-NMR (400 MHz, DMSO-*d_6_*): *δ* 17.17 (s, 1H, OH), 10.00 (bs, 4H, NH), 7.18 (m, 4H, Ph), 5.93 (s, 1H, benzyl-H), 2.88 (q, 4H, *J* = 7.3 Hz, C*H*_2_CH_3_), 1.12 (t, 6H, *J* = 7.3 Hz, CH_2_C*H*_3_); ^13^C-NMR (100 MHz, DMSO-*d_6_*): *δ* = 164.7, 164.0, 151.2, 144.6, 133.5, 129.9, 129.1, 127.8, 91.3, 42.1, 30.7, 11.8; LC/MS (ESI): 451[M]^+^; Anal. for C_19_H_22_ClN_5_O_6_; Calcd C, 50.50; H, 4.91; Cl, 7.85; N, 15.50; Found: C, 50.51; H, 4.90; Cl, 7.83; N, 15.51.

#### 3.1.13. 5,5′-((4-Methoxyphenyl)methylene)bis(6-hydroxypyrimidine-2,4(1*H*,3*H*)-dione) Diethylaminium Salt **4d**

**4d** was prepared from barbituric acid **1b**, and *p*-methoxybenzaldehyde **2f** according to the general procedure (**GP1**) yielding a beige powder (1.22 g, 2.73 mmol, 91%); m.p.: 195 °C; IR (KBr, cm^−1^): 3449, 3190, 2991, 2835, 1688, 1592, 1505, 1383, 1247; ^1^H-NMR (400 MHz, DMSO-*d_6_*): *δ* 17.26 (s, 1H, OH), 9.99 (bs, 4H, NH), 6.92 (d, 2H, *J* = 8.0 Hz, Ph), 6.72 (d, 2H, *J* = 8.0 Hz, Ph), 5.88 (s, 1H, benzyl-H), 2.90 (q, 4H, *J* = 7.3 Hz, C*H*_2_CH_3_), 1.14 (t, 6H, *J* = 7.3 Hz, CH_2_C*H*_3_); ^13^C-NMR (100 MHz, DMSO-*d_6_*): *δ* = 164.6, 164.0, 157.0, 151.2, 137.2, 132.4, 115.1, 91.7, 55.4, 42.1, 30.7, 11.6; LC/MS (ESI): 447[M]^+^; Anal. for C_20_H_25_N_5_O_7_; Calcd C, 53.69; H, 5.63; N, 15.65; Found: C, 53.69; H, 5.63; N, 15.66.

#### 3.1.14. 5,5′-(Naphthalen-2-ylmethylene)bis(6-hydroxypyrimidine-2,4(1*H*,3*H*)-dione) Diethylaminium Salt **4e**

**4e** was prepared from barbituric acid **1b**, and 2-naphthaldehyde **2i** according to the general procedure (**GP1**) yielding a beige powder (1.3 g, 2.79 mmol, 93%); m.p.: 192 °C; IR (KBr, cm^−1^): 3459, 3208, 2994, 1677, 1579, 1448, 1386, 1354; ^1^H-NMR (400 MHz, DMSO-*d_6_*): *δ* 16.92 (s, 1H, OH), 10.41 (bs, 4H, NH), 8.13 (d, 1H, *J* = 8.8 Hz, naphthyl), 7.81(d, 1H, *J* = 8.8Hz, naphthyl), 7.63 (d, 1H, *J* = 8.8 Hz, naphthyl), 7.38–7.32 (m, 4H, naphthyl), 6.46 (s, 1H, benzyl-H), 2.79 (q, 4H, *J* = 7.3 Hz, C*H*_2_CH_3_), 1.08 (t, 6H, *J* = 7.3 Hz, CH_2_C*H*_3_); ^13^C-NMR (100 MHz, DMSO-*d_6_*): *δ* = 164.9, 151.1,141.5, 135.8, 134.0,132.4, 129.3, 128.7, 126.0,125.8, 125.5, 125.2, 124.9, 123.8, 92.3, 42.5, 29.7, 12.7; LC/MS (ESI): 467[M]^+^; Anal. for C_23_H_25_N_5_O_6_; Calcd C, 59.09; H, 5.39; N, 14.98; Found: C, 59.12; H, 5.40; N, 15.01.

## 4. Conclusions

In summary, we have shown that the multicomponent tandem Aldol condensation/Michael addition reaction of aromatic aldehydes with barbituric acid **1a**,**b** by aqueous diethylamine medium is a powerful and efficient method for the synthesis of novel bis-pyrimidine derivatives **3a**–**i** and **4a**–**e**, which are of biological significance. In addition to its efficiency, simplicity and milder reaction conditions, this method provides excellent yields of the products with selectivity. Further studies on expanding the application of this method and the biological evaluation of these pyrimidine derivatives are in progress.

## Figures and Tables

**Table 1.** Screening of conditions for the Aldol-Michael addition reaction of model substrate a.

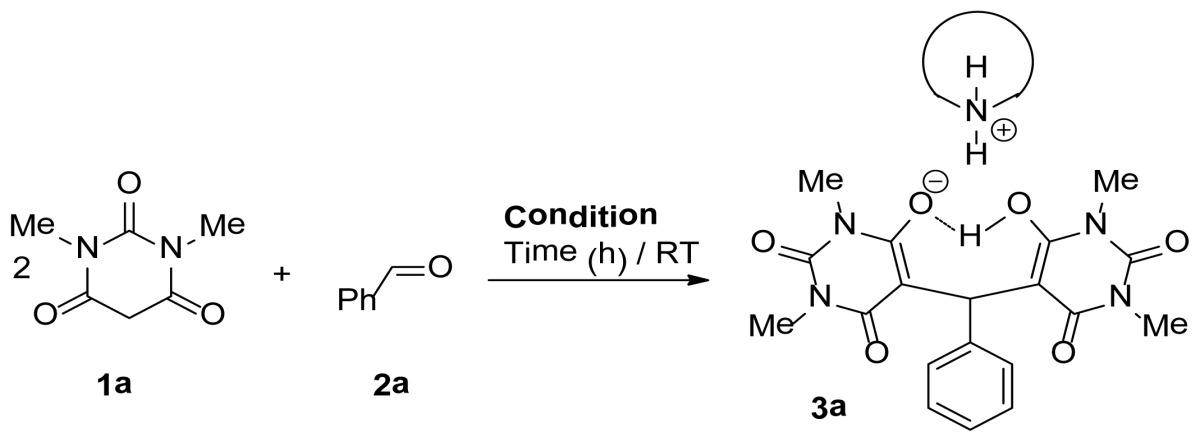			

Entry	Condition	Time (h)	Yield (%) b
1	Et_2_NH/H_2_O	1	99
2	*i*Pr_2_NH/H_2_O	5	85
3	(Cyclohexyl)_2_NH/H_2_O	4	82
4	Morpholine/H_2_O	3	78
5	NaOH/H_2_O	8	65
6	Et_2_NH	12	10
7	H_2_O	12	0

a All reactions were carried out with 1,3-dimethylbarbituric acid **1a** (3 mmol), benzaldehyde **2a** (1.5 mmol) and amine (1.5 mmol) in water (1.5 mL) for the specified time;

b Yield of isolated product.

**Table 2.** Tandem Aldol-Michael reactions of 1,3-dimethylbarbituric acid **1a** with aldehydes in aqueous diethylamine medium a.

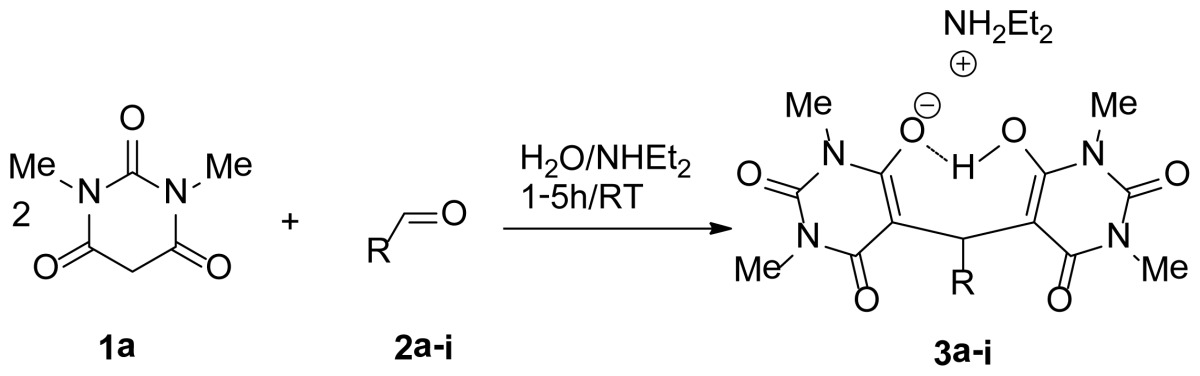			

Entry	3	R	Yield (%) b
1	**3a**	Ph	99
2	**3b**	*p*-CH_3_Ph	97
3	**3c**	*p*-ClPh	95
4	**3d**	*p*-BrPh	92
5	**3e**	*m*-BrPh	92
6	**3f**	*p*-CH_3_OPh	90
7	**3g**	*p*-NO_2_Ph	88
8	**3h**	*m*-CH_3_Ph	92
9	**3i**	2-Naphthaldehyde	94

a All reactions were carried out with 1,3-dimethyl barbituric acid **1a** (3 mmol), aldehydes **2a–i** (1.5 mmol) and amine (1.5 mmol) in water (1.5 mL) for the specified time;

b Yield of isolated product.

**Table 3.** Tandem Aldol-Michael reactions of barbituric acid **1b** with aldehydes in aqueous diethylamine medium a.

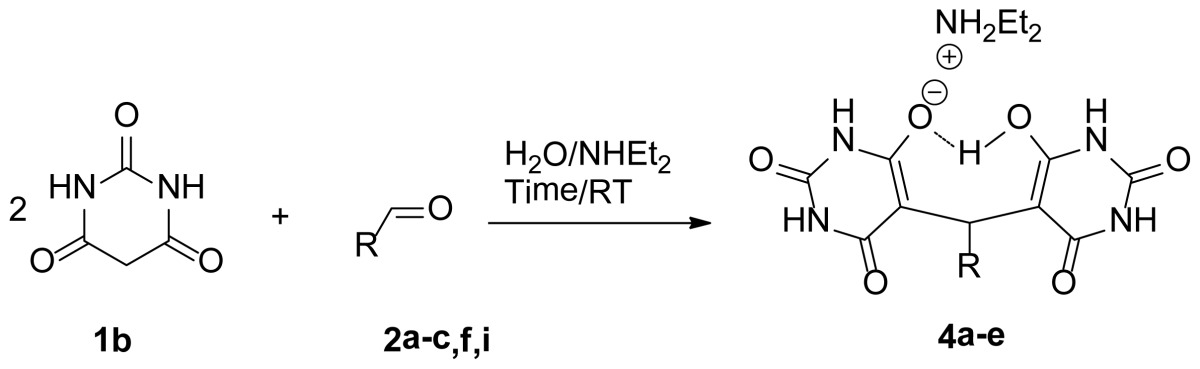			

Entry	4	R	Yield (%) b
1	**4a**	Ph	98
2	**4b**	*p*-CH_3_Ph	95
3	**4c**	*p*-ClPh	95
4	**4d**	*p*-CH_3_OPh	91
5	**4e**	2-Naphthaldehyde	93

a All reactions were carried out with barbituric acid **1b** (3 mmol), aldehydes **2** (1.5mmol) and amine (1.5mmol) in water (1.5 mL) for the specified time;

b Yield of isolated product.
