# Correction: Al-Majid, A.M., *et al.*, Tandem Aldol-Michael Reactions in Aqueous Diethylamine Medium: A Greener and Efficient Approach to Bis-Pyrimidine Derivatives. *Int. J. Mol. Sci*. 2013, *14*, 23762–23773.

**DOI:** 10.3390/ijms15057537

**Published:** 2014-04-30

**Authors:** Abdullah M. Al-Majid, Assem Barakat, Hany J. AL-Najjar, Yahia N. Mabkhot, Hazem A. Ghabbour, Hoong-Kun Fun

**Affiliations:** 1 Department of Chemistry, Faculty of Science, King Saud University, P.O. Box 2455, Riyadh 11451, Saudi Arabia; E-Mails: amajid@ksu.edu.sa (A.M.A.-M.); hany_33@hotmail.com (H.J.A.-N.); yahia@ksu.edu.sa (Y.N.M.); 2 Department of Chemistry, Faculty of Science, Alexandria University, P.O. Box 426-Ibrahimia, Alexandria 21321, Egypt; 3 Department of Pharmaceutical Chemistry, Faculty of Pharmacy, King Saud University, P.O. Box 2457, Riyadh 11451, Saudi Arabia; E-Mails: ghabbourh@yahoo.com (H.A.G.); hfun.c@ksu.edu.sa (H.-K.F.)

The authors wish to change Figure 2 in Section 2 of their paper published in *IJMS* [1]. Figure 2 is revised as follows. The authors and publisher apologize for any inconvenience.

**Figure 2.**
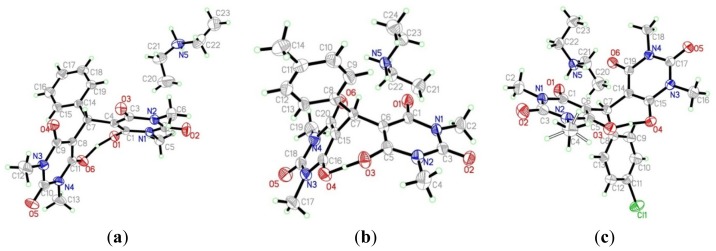
ORTEP representation of the structure of **3a**–**c**.
